# Postcraniotomy Headache: Etiologies and Treatments

**DOI:** 10.1007/s11916-022-01036-8

**Published:** 2022-03-01

**Authors:** Corina Bello, Lukas Andereggen, Markus M. Luedi, Christian M. Beilstein

**Affiliations:** 1 Department of Anaesthesiology and Pain Medicine, Inselspital, Bern University, Hospital, University of Bern, Freiburgstrasse, 3010, Bern, Switzerland; 2 Department of Neurosurgery, Kantonsspital Aarau, Aarau, Switzerland

**Keywords:** Postcraniotomy headache, Neurosurgery, Acute pain, Chronic pain, Enhanced recovery after surgery

## Abstract

**Purpose of Review:**

Postcraniotomy headache (PCH) is a highly underappreciated and very common adverse event following craniotomy.

**Recent Findings:**

Analgetic medication with opioids often interferes with neurologic evaluation in the acute phase of recovery and should be kept to a minimal, in general, in the treatment of chronic pain as well. We provide an update on the latest evidence for the management of acute and chronic PCH.

**Summary:**

Especially in the neurosurgical setting, enhanced recovery after surgery protocols need to include a special focus on pain control. Patients at risk of developing chronic pain must be identified and treated as early as possible.

## Background

Anaesthesia for craniotomies requires well established interdisciplinary teamwork. The patient population is very diverse, as is the age distribution, ranging from paediatric to geriatric patients, and their respective diseases among others with brain tumors, aneurysms or stroke. In order to obtain the precision required for surgical procedures in the brain tissue, optimal planning and anaesthetic management are needed. Adequate sedation and pain relief enable a timely return to consciousness for adequate neurologic evaluation. The usual pain medication—generally opioids—can interfere with this process.

Postoperative pain is still very common today and requires a multimodal analgetic approach to pain management. Especially in the field of neuroanaesthesia, sound data is still scarce. We aim to provide an update on the most recent evidence regarding the management of acute and chronic headache attributed to craniotomy.

Postcraniotomy headache (PCH) is very common. Moderate to severe pain has been reported in up to 60 to 90% of patients undergoing craniotomy [1, 2]. In addition, 30% of PCH patients suffer from chronic PCH, with a tremendous effect on quality of life, especially in the very young as well the frail population [3]. Besides the major impact it has on daily life after discharge, PCH also influences in-hospital recovery. Pain causes high blood pressure, which can lead to an increased risk of intracranial bleeding and intracranial hypertension. These factors not only prolong hospital stay, but also increases mortality along with health care costs [4].

Headache attributed to craniotomy is defined by the Committee of the International Headache Society as PCH within 7 days after surgical craniotomy and lasting for less than 3 months [5]. If it persists for more than 3 months, it is considered persistent or chronic. However, there is an ongoing debate over whether the definition of acute postoperative pain should be extended to include onset 30 days after craniotomy instead of only 7 [6]. Interestingly, the occurrence of postoperative pain highly depends on the surgical approach being chosen [3].

**Fig. 1:**
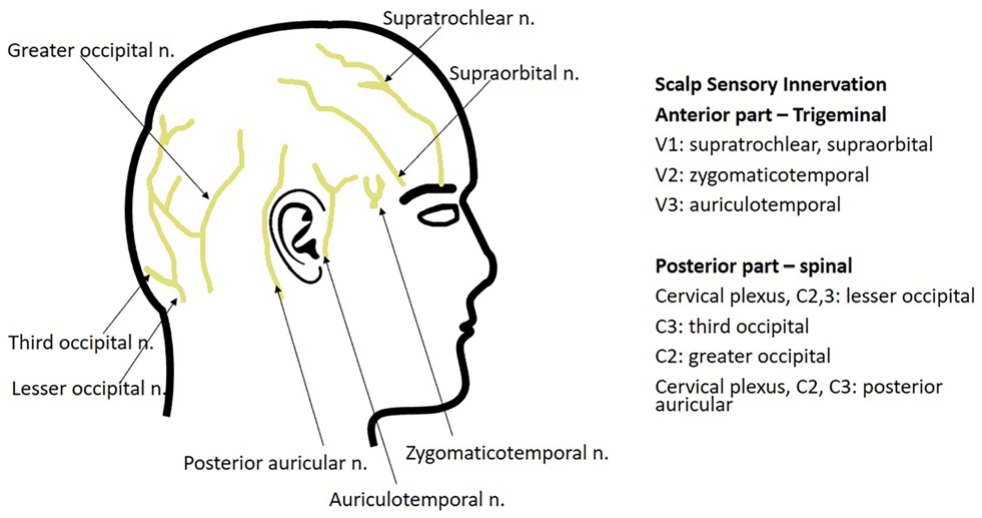
Scematic of scalp innervation

The brain tissue itself does not have pain sensors, but intracranial pressure can cause dural irritation and subsequently trigger pain. Thus, while in many patients with intracranial lesions headache is not infrequently reported, response to surgery can be beneficial [7–9]. However, the scalp is innervated by cranial nerves, rami spinales dorsales and ventral rami (Fig. 1). The trigeminal, occipital, vagus and hypoglossus nerves also supply some parts of the head.

## Risk Factors for Postcraniotomy Headache

There is evidence that there are multifactorial reasons for PCH, including damage of the nerve branches but also the formation of neuromas [10], nerve entrapment in the scar, traction of the dura, formation of adhesions (from dura to bone, dura to muscle or dura to brain) or muscle incision. Aseptic meningitis and intracranial hypotension due to a cerebrospinal fluid leak are special types of acute postoperative complications that need to be ruled out to diagnose craniotomy-attributed headaches.

Other risk factors apart from location and size of the incision include young age, female gender, preoperatively existent pain, anxiety and depression [11], all of which could be addressed in the preoperative anaesthetic assessment.

Performing a broad risk assessment in every individual is of great importance. Depression, chronic pain (sensitivity) and anxiety are factors that have a great influence on postoperative pain. Patient education can greatly improve the experience of pain by adequate information about the pain they have to expect. Patient history—including medications such as anti-coagulants, glucocorticoids and anti-convulsants—needs to be assessed, and premedication, including gabapentin or acetaminophen, might be prescribed. These can have an opioid-sparing effect, lessen the occurrence of delirium, improve sleep quantity and quality preoperatively, and lower the risk of postoperative nausea and vomiting (PONV). However, gabapentin may prolong the time to extubation and increase sedation requirements.

## Enhanced Recovery After Surgery

As in other high-risk settings where protocols for enhanced recovery after surgery (ERAS) have been set up, preoperative patient evaluation needs to play a key role [12•]. There is strong evidence suggesting that preoperative quantitative sensory testing (QST) might help identify patients at risk of developing acute and chronic postoperative pain. Most techniques are easy to perform and should be implemented in a preoperative standardised protocol, just as routine neurologic, cardiac, pulmonary and laboratory exams have been standardised [13].

Only after solid preparation for the procedure and prehabilitation of the patient can intraoperative management be optimally coordinated, with the goal of reducing the risk of PCH. The choice of anaesthesia technique still depends on individual factors; however, intravenuous agents might be a better option when compared to inhalational agents such as sevoflurane, due to their better risk profile concerning haemodynamic stability, PONV [14] and inflammatory effects, all of which have an impact on postoperative pain [15, 16].

Adequately controlling pain while maintaining safety is very complex in operative neurosurgical procedures. Commonly used opioids such as fentanyl have many unwanted side effects, including sedation, nausea, vomiting and miosis. These can interfere with proper neurological exams postoperatively. Long-acting opioids such as fentanyl can interfere with intraoperative and postoperative neuro-monitoring [17]. Therefore, shorter-acting agents such as remifentanil might allow for better neurological assessment than long-acting opioids. However, postemergence hyperalgesia is a common problem with remifentanil.

Some other medications might have a superior side effect profile. Paracetamol provides analgesia without sedation. Therefore, this agent would be good for clinical evaluation, but alone does not provide enough potency to mitigate pain. Non-steroidal anti-inflammatory drugs (NSAIDs) are commonly used pain medications that come in two distinct forms. Non-selective cyclooxygenase (COX) 1 and 2 inhibitors have a negative side effect profile including haemorrhage, renal failure and peptic ulcers [18]. The increased risk of bleeding and seizures can both lead to harmful events in neurosurgical procedures. Alternatively, selective COX2 inhibitors lead to less bleeding but seem to lack an opioid-sparing effect, limiting the tendency to switch to such costly options [19, 20]. Lastly, metamizol might also be of interest but has an associated risk of drug-induced liver injury [21], and agranulocytosis and is therefor not recommended in every institution or patient setting.

Adjuvants such as lidocaine or dexmedetomidine can be used to spare opioids [22, 23, 24•].

Ketamine has been shown to improve cerebral perfusion; however, there is still a lack of evidence concerning the neurologically relevant side effects, such as cognitive disturbances, dizziness, visual problems and hallucinations [25]. When the side effects are severe, they can have a tremendous effect on this high-risk neurologic population, leading to limits on the use of ketamine as an adjuvant analgesic. Corticosteroids have been widely used for PONV prophylaxis. Their effect on pain is still unknown, but there are some studies showing benefits for pain management [26]. In glioblastomas, dexamethasone has been shown to have an oncogeneic effect on cancer cells, and therefore needs to be used with caution in this patient population [27•, 28]. In supratentorial surgery, lidocaine infusions have been tested and shown to have a positive effect in preventing postoperative pain. Finally, a sodium channel blocker generally used for the treatment of non-surgically related headache might also be beneficial in the treatment of acute PCH [29–31].

## Multimodal and Interdisciplinary Approaches

Modern anaesthesia calls for a multimodal approach. Regional anaesthetic techniques are widely used in other specialties, such as abdominal wall surgical procedures, where local anaesthetic is inserted intrafascially in order to relieve pain postoperatively [32]. In craniotomy, scalp infiltration with a local anaesthetic is a promising technique. It has been shown to decrease the risk of bleeding when epinephrine is added to the local anaesthetic. Additionally, it has a positive impact on local haemodynamic response to skull fixation—for example, in the Mayfield frame—and is thereby effective in preventing persistent neuropathic pain. However, no benefit for acute postoperative pain has been found [17].

Direct singular nerve blocks can also be used for craniotomy and is standard in patients undergoing awake surgery along with intraoperative cortical stimulation. Such blocks are generally done “blindely”, although ultrasound guidance is already standard for all other truncal and peripheral blocks [33]. Scalp nerves include supraorbital, auriculotemporal, occipital or zygomatico-temporal nerve branches. This technique has been shown to be more promising for acute postoperative pain than scalp infiltration. However, intraoperatively, there has not been any effect on anaesthetic requirements. Moreover, severe bradycardia was reported by Chowdhury et al. [34] after a trigeminal nerve block mitigated via trigeminocardiac reflex, pointing out the importance of close monitoring of vital parameters and evaluation of patient risk factors.

The application of subcutaneous sumatriptan is another technique which is still under investigation but might be promising [35].

Finally, an interdisciplinary approach might be the best solution to manage acute postoperative pain after all. As the surgeons work directly on the scalp, they can infiltrate the wound locally along the scar. The size of the incision is highly associated with the intensity of pain. Evidence assessing the effectiveness of postoperative compared to preoperative application of local infiltration of the wound is scarce; however, better emergence has been reported if the infiltration happens right before the end of the surgery, again showing the necessity for clear communication between the surgeon and the anaesthetist.

In the special setting of awake craniotomy, other promising techniques such as cingulum stimulation have been tested and shown to be effective in managing scalp pain intraoperatively [36]. Such techniques are highly efficient in providing adequate pain relief in the awake setting. There are minimal interacting side effects when it comes to neurologic evaluation of the patient. Postoperatively, however, there is a lack of evidence with regards to such techniques.

In the postoperative setting, there have been many studies discussing different approaches to managing PCH. Non-pharmacological treatments include cool packs, cryotherapy or head dressings [6]. The use of opioids produces the same problems already discussed in the intraoperative setting. There are case reports of methadone used as a primary analgesic agent with very good results in terms of pain control and side effects [37]. Patient-controlled analgesia (PCA) is a highly efficient method to provide adequate pain control. Nowadays, morphine has the best safety profile for postoperative use in this special setting [38, 39]. Some other studies using fentanyl as the analgesic agent in PCA have shown beneficial effects due to its shorter half-life compared to morphine [40]. NSAIDs might still offer a good additive choice to control postoperative pain. Nontheless, involving the whole care team and creating a solid pain evaluation and management plan is key to minimizing the development of chronic pain.

The pathophysiologic mechanisms behind chronic pain are complex, and on a molecular level remain unclear. There is evidence showing sensitisation of central neurologic pain perception and structural changes in receptive fields of neurons in the central nervous system. Migraine- or tension-like headaches are associated with hyperstimulation of GABA receptors in the raphe nuclei and changes in the serotonergic and haemodynamic systems [41]. Catecholaminergic nerves might also play a key role in patients suffering from chronic pain [42, 43].

In neurosurgery, the outcome of postoperative chronic pain is highly dependent on the preoperative assessment. Promising techniques such as QST are a great way to identify patients at risk [13]. Pressure pain threshold measurements can help assess patients at risk of developing chronic PCH [44]. Such early recognition of a population at risk helps focus discussion on adequate early treatment and multiple pain—desensitising options to actively stop or even prevent the development of PCH.

Pharmacologic treatment modalities for chronic PCH include tricyclic drugs such as amitriptyline [45], as well as anti-convulsants. Valproate might help in migraine-like headaches [30], whereas gabapentin has been shown to be effective for neuropathic cranial pain [46, 47]. Carbamazepine [48] and lamotrigine [49] are other potential agents. Injection of botox for tension-type headache has been tried and showed beneficial effects in most chronic PCH patients [50, 51]. The use of opioids—while very effective over the short term—should be limited considering the rapid increase in opioid-dependant patients worldwide [52].

Other promising pharmacological interventions which are under current investigation include drugs targeting NR2B-subunit-selective N-methyl-D-aspartate receptors [53], voltage-gated sodium channels (VGSC) or tetrodotoxin-receptors, all of which play an important role in the development of chronic pain. However, pharmacologic evidence for such agents is still in its infancy.

## Implementation in Clinical Practice

The patient population undergoing craniotomy includes a wide range of generations, starting already at a very young age. Managing pain in children is of great importance, and fast recovery is particularly warranted. The development of chronic PCH as defined by the International Headache Society might not be as common in this population as in the elderly [54], but still, PCH occurs in 42% of children within the first 72 h [55]. Especially in this population, opioid use is an important problem. Overall, there is a decreasing tendency toward self-reported opioid use in the paediatric population, but dependance on opioids is still problematic [56].

NSAIDs are very widely used postoperatively in children, and have been shown to be safe and to provide adequate analgesia [57]. Just as in adult patients, morphine-PCA is very promising in the acute setting [58]. There are studies evaluating the efficacy of ropivacaine for local scalp nerve blocks in the paediatric population, but there are still no results [59]. Other trials are assessing potential benefits of adding prednisolone to preemptive local ropivacaine scalp infiltrations [60]. There seems to be a local anti-inflammatory and anti-swelling effect without an oncogenic effect on glioblastomas [27•, 28] of orally applied glucocorticoids such as dexamethasone [26] (Tables 1 and 2).

**Table 1:** Managing acute post-craniotomy headache

**Type of treatment**	**Evidence**	**Caveat**	**Reference**
Codeine	With paracetamol but not alone	Metabolization	Sudheer et al. [61], Goldsack et al. [62], Jeffrey et al. [63]
Morphine, long-acting opioids	Morphine superior to other opioids; hydromorphine may be better	Cerebral circulation and metabolism impaired…	Sudheer et al. [61], Cold et al. [64]
Tramadol	Good for acute postoperative pain	Side effects (PONV; drowsiness), less efficient than morphine	Jeffrey et al. [63]
PCA	Very good with MO or fentanyl for pain control, PONV, sedation		Morad et al. [65]
NSAID – non-selective			Kelly et al. [66]
Ketamine	Improves cerebral perfusion intraoperatively	Cognitive disturbances, dizziness, visual problems, hallus, effect on pain unclear	Mayberg et al. [14], Markovic-Bozic et.al. [15], Himmelseher et al. [25], Misra et al. [26]
Lidocaine infusion	Postop acute pain reduced		Peng et al. [22]
Others	Gabapentin (for better sleep and neuropathic pain), amitriptyline (tension headache chronic), valproate (migraine-like), carbamazepine (chronic tension–like), lamotrigine (neuralgia)		Silberstein et al. [30], Moore et al. [51], Karst et al. [48], Sandner et al. [49]
NR2B-subunit-selective N-methyl-D-aspartate receptor antagonists; tetrodotoxin, VGSC	For prevention of chronic headache		Perucca et al. [53]

*NSAIDs* non-steroidal anti-inflammatory drugs, *PCA* patient-controlled analgesia, *PONV* postoperative nausea and vomiting, *VGSC* voltage-gated sodium channels

**Table 2:** Post-craniotomy headache in children

**Author**	**Year**	**Study type**	**Arms**	**Anaesthesia maintenance technique**	**Opioid use (intraop and postop)**	**Postop findings**
Xing et al. [58]	2019	Randomised controlled trial, 320 children 1–12 yr	Control group normal saline 100 ml, 2 ml/h, bolus 0.5 ml; fentanyl 0.1–0.2 μg/k·h, bolus 0.1–0.2 μg/kg; morphine 10–20 μg/kg·h, bolus 10–20 μg/kg; tramadol 100–400 μg/kg·h, bolus 100–200 μg/kg	Remifentanyl and sevoflurane	Rescue medication: ibuprofen, morphine	PCIA, NCIA with morphine safe and most effective (less postoperative pain, no increase in PONV, respiratory depression, sedation), most nausea in tramadol, less pain in fentanyl and tramadol, risk factors for moderate to severe pain: young children, occipital craniotomy, morphine treatment
Nesvick et al. [57]	2020	Retrospective cohort study	276 patients under 18 yr of age			NSAID on postop day 1 do not increase postoperative haemorrhage requiring return to the operating room or incidence of more-than-minimal haemorrhage on routine postoperative imaging
Xiong et al. [59]	2020	Prospective, randomised, placebo-controlled, double-blind trial	180 patients age 1–12	Preoperative scalp nerve block with ropivacaine, postoperative block, no block	Sufentanil	Under investigation – primary outcome: pain score, consumption of sufentanil within 24 h, additive analgesic agents, length of hospital stay, complications
Zhao et al. [60]	2019	Prospective randomized controlled trial	100 patients aged 8–18	Scalp nerve block with ropivacaine + methylprednisolone, scalp nerve block with ropivacaine only		Under investigation – primary outcome: cumulative PCA-fentanyl-dose within 24 h; secondary outcome: postoperative Numerical Rating Scale scores, pain control satisfaction scores, length of stay and adverse events

*NSAIDs* non-steroidal anti-inflammatory drugs, *PCA* patient-controlled analgesia

## Conclusion

In summary, PCH is a highly underappreciated adverse event following craniotomy. Especially in the neurosurgical setting, ERAS protocols need to include a special focus on pain control. The aim should be to identify the population at risk of developing chronic pain conditions. Just as in other high-risk specialties such as cardiac surgery, a multimodal and interdisciplinary approach is a must. Specific methods to prevent transition from acute to chronic PCH include the preoperative identification of risk factors (depression, chronic pain and anxiety) with initiation of respective pharmacological therapy as soon as possible. In addition, acute pain after craniotomy must be managed proactively, to minimize the risk of transition to chronic pain. More evidence is needed, however, to define which anaesthetic techniques best serve the patient while allowing safe neurologic evaluation and providing adequate pain control. This will enable treating physicians to provide optimal care in this very diverse patient population.

## Data Availability

Not applicable.

## Abbreviations

COX: Cyclooxygenase
ERAS: Enhanced recovery after surgery
NSAIDs: Non-steroidal anti-inflammatory drugs
PCA: Patient-controlled analgesia
PCH: Postcraniotomy headache
PONV: Postoperative nausea and vomiting
QST: Quantitative sensory testing
TENS: Transcutaneous electric nerve stimulation
VGSC: Voltage-gated sodium channels
